# A comprehensive metabolic profiling of the metabolically healthy obesity phenotype

**DOI:** 10.1186/s12944-020-01273-z

**Published:** 2020-05-09

**Authors:** Vibeke H. Telle-Hansen, Jacob J. Christensen, Gulla Aase Formo, Kirsten B. Holven, Stine M. Ulven

**Affiliations:** 1 Faculty of Health Sciences, Oslo Metropolitan University, P.O. Box 4, St. Olavsplass, 0130 Oslo, Norway; 2 Norwegian National Advisory Unit on Familial Hypercholesterolemia, Oslo University Hospital Rikshospitalet, P.O. Box 4950, Nydalen, 0424 Oslo, Norway; 3 Departmentof Nutrition, Institute of Basic Medical Sciences, Faculty of Medicine, University of Oslo, P.O. Box 1046, Blindern, 0317 Oslo, Norway

**Keywords:** Metabolically healthy obesity, Metabolically unhealthy obesity, Fatty acids, SCFA, Obese, Diet, Glycemic regulation, Lipoprotein, Metabolic profiling

## Abstract

**Background:**

The ever-increasing prevalence of obesity constitutes a major health problem worldwide. A subgroup of obese individuals has been described as “metabolically healthy obese” (MHO). In contrast to metabolically unhealthy obese (MUO), the MHO phenotype has a favorable risk profile. Despite this, the MHO phenotype is still sub-optimally characterized with respect to a comprehensive risk assessment. Our aim was to increase the understanding of metabolic alterations associated with healthy and unhealthy obesity.

**Methods:**

In this cross-sectional study, men and women (18–70 years) with obesity (body mass index (BMI) ≥ 30 kg/m^2^) or normal weight (NW) (BMI ≤ 25 kg/m^2^) were classified with MHO (*n* = 9), MUO (*n* = 10) or NW (*n* = 11) according to weight, lipid profile and glycemic regulation. We characterized individuals by comprehensive metabolic profiling using a commercial available high-throughput proton NMR metabolomics platform. Plasma fatty acid profile, including short chain fatty acids, was measured using gas chromatography.

**Results:**

The concentrations of very low density lipoprotein (VLDL), intermediate density lipoprotein (IDL) and low density lipoprotein (LDL) subclasses were overall significantly higher, and high density lipoprotein (HDL) subclasses lower in MUO compared with MHO. VLDL and IDL subclasses were significantly lower and HDL subclasses were higher in NW compared with MHO. The concentration of isoleucine, leucine and valine was significantly higher in MUO compared with MHO, and the concentration phenylalanine was lower in NW subjects compared with MHO. The fatty acid profile in MHO was overall more favorable compared with MUO.

**Conclusions:**

Comprehensive metabolic profiling supports that MHO subjects have intermediate-stage cardiovascular disease risk marker profile compared with NW and MUO subjects.

**Clinical trial registration number:**

NCT01034436**,** Fatty acid quality and overweight (FO-study).

**Graphical abstract:**

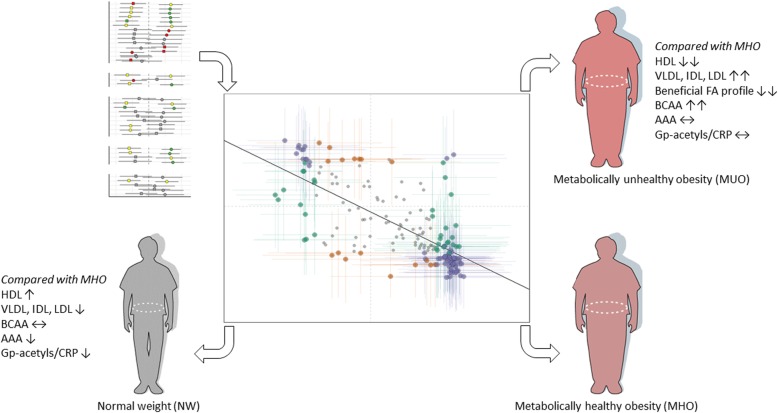

## Background

Obesity alters the state of metabolism and physiology leading to dyslipidemia, insulin resistance, and inflammation, and is therefore an important risk factor for cardiovascular diseases (CVD) and type 2 diabetes (T2D) [1]. Furthermore, estimates show that every 5 units higher body mass index (BMI) above 25 kg/m^2^ is associated with about 31% higher risk of premature death [2]. Prevention and treatment of obesity and obesity-related diseases are therefore major public health challenges which needs to be solved. However, obesity is a heterogeneous and complex condition and a subgroup of individuals with obesity has been described to have “metabolically healthy obesity” (MHO) [3]. In contrast to metabolically unhealthy obesity (MUO), the MHO phenotype has a favorable lipid profile and a normal or only slightly affected insulin sensitivity, despite the similar amount of body fat [3]. Weight reduction per se will improve metabolic risk factors, but is difficult both to achieve and to maintain for a longer period. Some studies have reported individuals with MHO to have an intermediate-stage risk of metabolic disorders compared with individuals with healthy, normal weight (NW) and MUO [4, 5], and that the MHO phenotype is associated with a higher risk of coronary heart disease and heart failure than a healthy, NW phenotype [5]. Even though individuals with MHO will shift towards an MUO profile with time, a more profound understanding of the underlying metabolic regulation in MHO and MUO is necessary to enhance our understanding of the development of metabolic dysfunction associated with obesity, and how to prevent it with lifestyle changes.

Several studies have investigated metabolites as biomarkers of metabolic dysregulation in obesity [6–8]. However, differences between subgroups of individuals with obesity, like MHO and MUO, are less investigated. A detailed study of lipoprotein metabolism and the detection of subtle differences in the distribution of lipoproteins between MHO and MUO may increase our understanding of the lipid metabolism in obesity, to target prevention and treatment more precisely among MUO and MHO.

## Methods

Our aim was to increase the understanding of metabolic alterations associated with healthy and unhealthy obesity. We hypothesized that for all features associated with the discrimination of obesity subtypes, 1) individuals with MUO would present with a more detrimental phenotype than MHO, and 2) MHO would present with a more detrimental phenotype than NW.

In this exploratory, cross-sectional study, we characterized individuals with MUO, MHO and NW by comprehensive metabolic profiling of the following systemic biomarkers in plasma: lipoprotein subclasses, glycolysis related metabolites, amino acids, ketone bodies, fluid balance, inflammatory markers, as well as fatty acid profile data and dietary intake data. The participants in this cross-sectional study was initially recruited to a dietary intervention study designed to investigate the effect of diglyceride oil on metabolic regulation [9], hence the data presented in the present article are considered explorative with a hypothesis generating purpose.

### Study population

The inclusion criteria for study participation were men and women with obesity (18–70 years, BMI ≥ 30 kg/m^2^) and has previously been described [10]. The participants were characterized with MHO (*n* = 9) when at least three out of the following five criteria were fulfilled: Homeostasis model assessment of insulin resistance (HOMAir) index ≤ 1.95, triglyceride (TG) ≤ 1.7 mmol/L, total cholesterol ≤ 5.2 mmol/L, low-density lipoprotein (LDL)-cholesterol ≤ 2.6 mmol/L, and high density lipoprotein (HDL)-cholesterol ≥ 1.3 mmol/L. Individuals with a MUO profile (*n* = 10) were characterized by fulfilling at least four out of the following five criteria: HOMAir index > 1.95; TG > 1.7 mmol/L; total cholesterol > 5.2 mmol/L; LDL-cholesterol > 2.6 mmol/L and HDL-cholesterol < 1.3 mmol/L. The criteria used in the present study are based on the National Cholesterol Education Program’s Adult Treatment Panel III report (ATP III) for lipid profiles as previously described by Karelis et al. [3]. Furthermore, eleven individuals with a healthy, NW phenotype (BMI ≤ 25 kg/m^2^) were included, characterized as healthy when at least four out of five of the MHO criteria were present.

The study was approved by the Regional Committee of Medical Ethics (approval no. 6.2008.1368) and by the Norwegian Social Science Data Services (approval no. 19667). Written informed consent for participation was obtained from each participant, and the study complied with the Declaration of Helsinki. The study was registered at Clinical trials (NCT01034436).

### Measurements of body composition

BMI and body composition were estimated by Tanita BC-418 bioelectric impedance analysis (BIA, 50 kHz), a hand-to-foot system, according to the manufacturer’s manual (Tanita Corporation, Tokyo, Japan), and has previous been described in Telle-Hansen et al. [10]. All participants were measured while standing in a relaxed position and with normal respiration. All participants were routinely classified as “standard” body type.

### Blood sampling

Participants were told to refrain from alcohol consumption and vigorous physical activity the day prior to blood sampling. Venous blood samples were drawn after an overnight fast (12 h). Serum was obtained from silica gel tubes [Becton–Dickinson (BD) vacutainer] and kept at room temperature for at least 30 min, until centrifugation (1500 g, 12 min). Serum was kept at room temperature and immediately prepared for subsequent analysis of routine laboratory analyses or aliquoted and stored at − 80 °C until further analyses. Plasma was obtained from EDTA tubes (BD vacutainer), immediately placed on ice and centrifuged within 10 min (1500 g, 4 °C, 10 min). Plasma samples were aliquoted and stored at − 80 °C until further analyses.

### Routine laboratory analysis

Fasting serum total cholesterol, LDL-cholesterol, HDL-cholesterol, TG, glucose, insulin, and HbA1c were measured by standard methods at Oslo University Hospital, Norway.

### Plasma fatty acid profile and short chain fatty acids

Fasting EDTA plasma fatty acid profile was measured with gas chromatography (GC-MS (short-chain fatty acids (SCFA)) and GC-FID (fatty acids)) using a commercial laboratory (Vitas Analytical Service, Oslo, Norway). The concentration of the individual fatty acids was measured as μg fatty acid/ml plasma and presented as percentage of total fatty acids.

### NMR spectroscopy

Metabolic biomarkers were quantified from fasting EDTA plasma using a commercially available high-throughput proton NMR metabolomics platform (Nightingale Health Ltd., Helsinki, Fin), giving a snapshot of systemic metabolism. This method quantifies lipoprotein subclass profile with lipid concentrations within fourteen subclasses, abundant proteins and various low-molecular-weight metabolites. Details of the experimentation and applications of the NMR metabolomics platform have been described previously [11]. The fourteen lipoprotein subclass sizes were defined by their average diameter, as follows: extremely large (XXL) very low density lipoprotein (VLDL)/chylomicrons (> 75 nm), extra-large (XL), large (L), medium (M), small (S), and extra-small (XS) VLDL (64.0, 53.6, 44.5, 36.8 and 31.3 nm), intermediate lipoprotein IDL (28.6 nm), L, M, and S LDL subclasses (25.5, 23.0 and 18.7 nm), and XL, L, M, and S HDL subclasses (14.3, 12.1, 10.9 and 8.7 nm). The following components of the lipoprotein subclasses were quantified: phospholipids (PL), cholesterol, cholesteryl esters (CE), free cholesterol (FC) and TG. The mean size for VLDL, LDL and HDL particles was calculated by weighting the corresponding subclass diameters with their particle concentrations.

### Dietary registration

All participants with obesity were invited to complete a four-day, pre-coded food diary, in which fifteen participants (6 MHO and 9 MUO) completed the registration. The diary included > 270 food items grouped together according to the typical Norwegian meal pattern [12]. Each food group was supplemented with open-ended alternatives. Along with the food diary, each participant received a validated photography booklet that contained thirteen series of colored photographs, each with four different portion sizes ranging from small to large. Food amounts were estimated in predefined household units (e.g. glasses, pieces or tablespoons) or from photographs. The diaries were scanned using the Teleform program, version 6.0 (InfoShare Solutions AS). Daily intake of energy and macronutrients was computed using the Norwegian food database and software system KBS (KBS, version 7), developed at the Department of Nutrition, University of Oslo, Norway.

### Statistics and bioinformatics analyses

#### Tools

All data analyses were performed in R version 3.6.0 using R Studio version 1.2.1335. In this section, we refer to packages and specific functions where relevant in the following format: *package::function (settings)*. Note that settings that deviate from the default are noted in parentheses.

### Exploratory data analyses

To get an impression of the data types and their unsupervised separation of group affiliation, we performed principal component analyses (PCA) for each data type (stats::prcomp (scale = TRUE)). Note that we normalized all variables prior to running the analysis. We visualized PC1 and PC2 in scatter plots, highlighting group and explained variance for each component.

### Pre-processing

To optimize downstream modeling, we pre-processed the data. All skewed variables (e1071::skewness (na.rm. = TRUE) lower than − 1 or higher than 1 were transformed using Box-Cox transformation (caret::BoxCoxTrans (na.rm. = TRUE)). Next, we normalized all variables (stats::scale), both un-transformed and transformed variables, to mean = 0 and SD = 1, making them directly comparable in the same downstream forest plot visualization. These pre-processing steps were performed for MHO and MUO combined, and for MHO and NW combined.

### Linear regression models

We performed linear models using stats::lm, and retrieved all relevant coefficients using broom::tidy (conf.int = TRUE). We compared MUO vs MHO and NW vs MHO in separate models, and we performed the analysis using various adjustment levels, including 1) no covariates, 2) age, 3) gender, 4) age and gender, and 5) age*gender (interaction). Since there was little or no discrepancy between the different adjustment levels, we report group estimates and associated uncertainty measures and *P* values adjusted for age and gender.

## Results

### Characteristics of the participants

Data from thirty participants (*n* = 18 males/12 females) was available for this study and the sample population has been described before [10]. The mean age was 49 years (range 42–63 years; MHO, *n* = 9), 52 years (43–59 years; MUO, *n* = 10) and 47 years (42–54 years; NW, *n* = 11) with a BMI of 33 (30–37 kg/m^2^), 32 (30–34 kg/m^2^) and 23 (21–24 kg/m^2^), respectively [10].

### Principal component analysis

First, we analyzed the variability among the groups using PCA by either clinical data, different metabolites (particle concentrations of lipoprotein subclasses, glycolysis related metabolites, amino acids, ketone bodies, fluid balance markers, and inflammatory markers), as well as plasma fatty acid composition and dietary intake (Additional file 1). The three groups were fairly well separated for the two former data types; MUO and MHO groups were also well separated by fatty acids but not by dietary intake. This indicates that all except the diet show an unsupervised ability to discriminate between the study groups.

### Particle concentration of lipoprotein subclasses

**Fig. 1:**
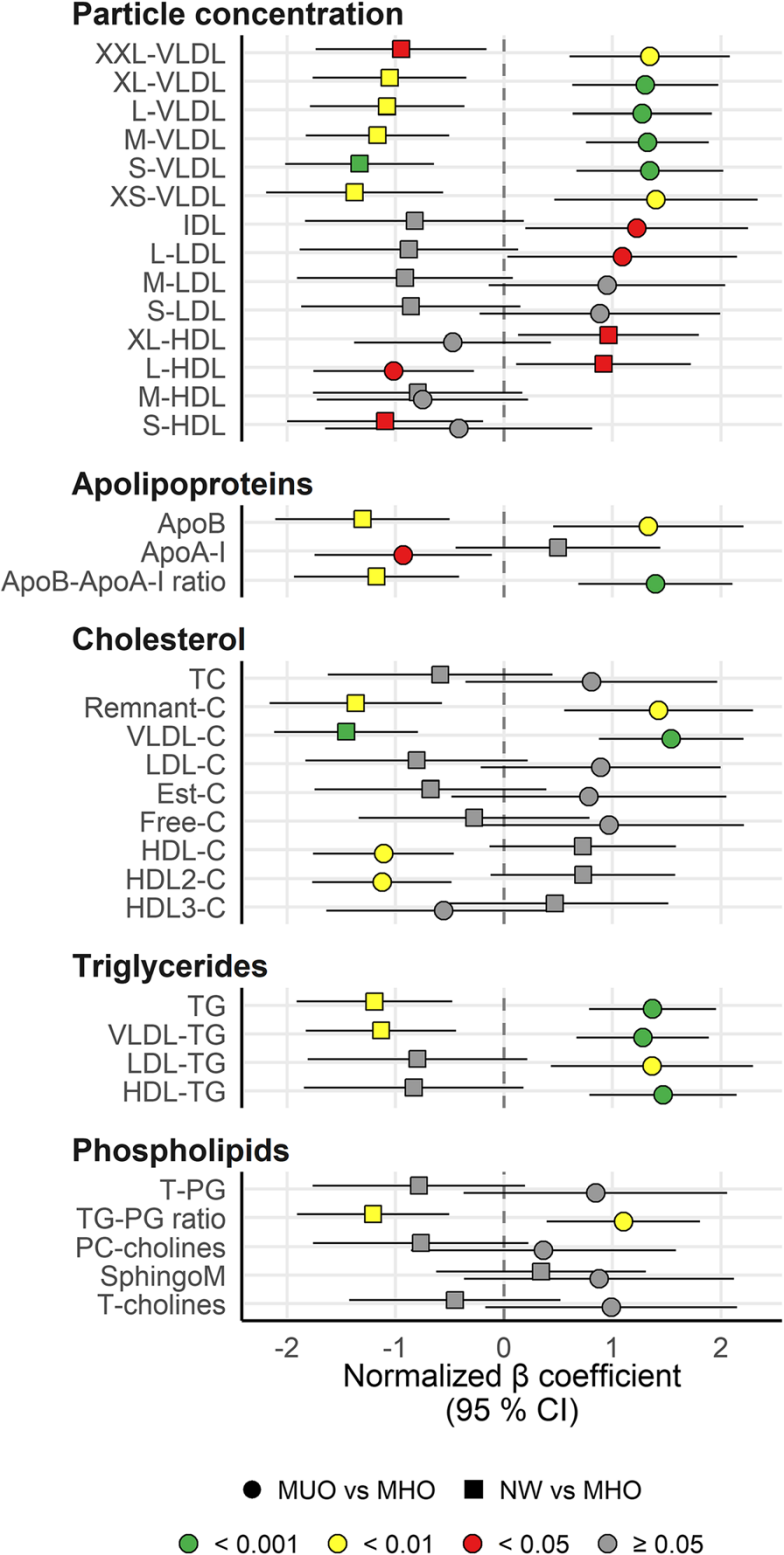
Atherogenic lipoprotein particles were increased in MUO, and reduced in NW, compared with MHO. The forest plot displays the β regression coefficients (mean difference) and 95% confidence interval for MUO vs MHO subjects (circles) and NW vs MHO subjects (squares). Estimates on the right and left side of the zero-line translates to *higher* and *lower* than MHO subjects, respectively. Color denotes nominal significance level. Abbreviations: ApoA-I, Apolipoprotein A-I; ApoB-ApoA-I ratio, Ratio of apolipoprotein B to apolipoprotein A-I; ApoB, Apolipoprotein B; Est-C, Esterified cholesterol; Free-C, Free cholesterol; HDL-C, Total cholesterol in HDL; HDL-TG, Triglycerides in HDL; HDL, High-density lipoprotein; HDL2-C, Total cholesterol in HDL2; HDL3-C, Total cholesterol in HDL3; IDL, Intermediate-density lipoprotein; L, Large; LDL-C, Total cholesterol in LDL; LDL-TG, Triglycerides in LDL; LDL, Low-density lipoprotein; M, Medium; MHO, Metabolically healthy obese subjects; MUO, Metabolically unhealthy obese subjects; NW, Normal weight subjects; PC-cholines, Phosphatidylcholine and other cholines; Remnant-C, Remnant cholesterol (non-HDL, non-LDL -cholesterol); S, Small; SphingoM, Sphingomyelins; T-cholines, Total cholines; T-PG, Total phosphoglycerides; TC, Serum total cholesterol; TG-PG ratio, Ratio of triglycerides to phosphoglycerides; TG, Serum total triglycerides; VLDL-C, Total cholesterol in VLDL; VLDL-TG, Triglycerides in VLDL; VLDL, Very low-density lipoprotein; XL, Extra-large; XS, Extra-small; XXL, Extremely large

Although individuals with MHO have a favorable clinical lipid phenotype based on the definition of MHO, their atherogenic lipoprotein profile is poorly characterized. Here we found that MUO and NW present with a more risk-prone and healthy atherogenic lipoprotein profile, respectively, compared with MHO (Fig. 1).

Due to the definition used to characterize the obesity phenotypes, the MUO and MHO groups would differ in cholesterol and TG levels; the lipoprotein profiling showed that this difference is mediated by significant higher or lower concentration of the whole spectrum of VLDL particles, IDL and L-LDL, ApoB, TG and PL, in the MUO and NW groups, compared with the MHO group, respectively. Interestingly, the largest HDL particles and ApoA-I followed the same pattern: they were significantly higher and lower in NW and MUO, respectively, compared with MHO.

Furthermore, the absolute level (but not relative level) of various lipid types (PL, total cholesterol, CE, FC and TG) in the different lipoprotein subclasses (VLDL, IDL, LDL and HDL) were overall higher in MUO and lower in NW, compared with MHO (Regression estimates in Additional file 2; raw data in Additional file 3).

### Amino acids and various biomarkers

**Fig. 2:**
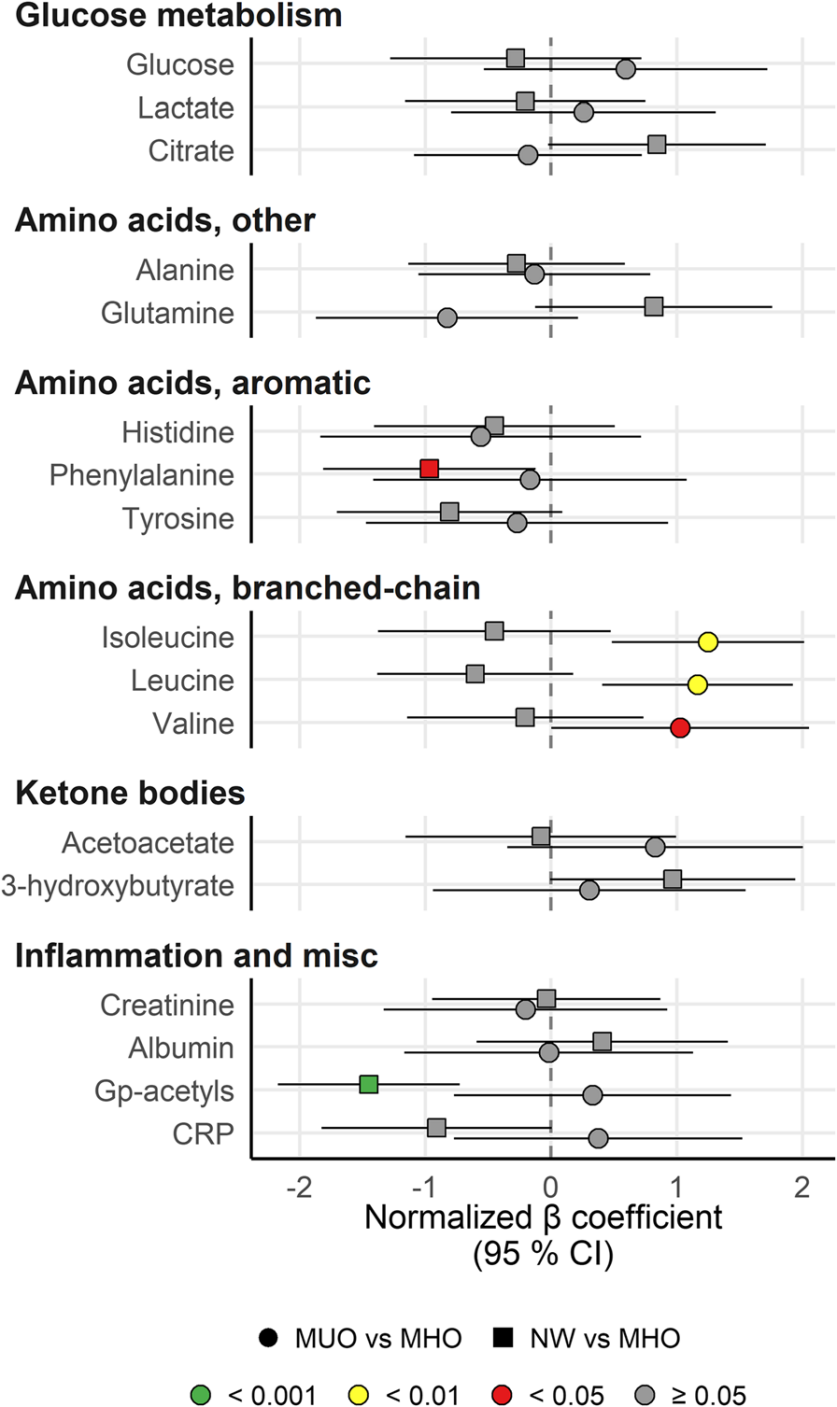
Branched-chain amino acids were generally higher in MUO vs MHO, whereas inflammation markers are lower in NW vs MHO. The forest plot displays the β regression coefficients (mean difference) and 95% confidence interval for MUO vs MHO subjects (circles) and NW vs MHO subjects (squares). Estimates on the right and left side of the zero-line translates to *higher* and *lower* than MHO subjects, respectively. Color denotes nominal significance level. Abbreviations: CRP, C-reactive protein; Gp-acetyls, Glycoprotein acetyls, mainly a1-acid glycoprotein; MHO, Metabolically healthy obese subjects; MUO, Metabolically unhealthy obese subjects; NW, Normal weight subjects

We found that the fasting concentrations of the branched chain amino acids (BCAA) isoleucine, leucine and valine were significantly higher in individuals with MUO compared with MHO (Fig. 2). In addition, the fasting concentration of phenylalanine was significantly lower in NW compared to MHO. Interestingly, NW had significantly lower level of Gp-acetyls and borderline significant lower C-reactive protein (CRP), indicating that both obese groups had ongoing low grade inflammation (Fig. 2). All other metabolites were statistically similar between the groups.

### Fatty acid profile in plasma and estimated stearoyl-CoA desaturase activity

**Fig. 3:**
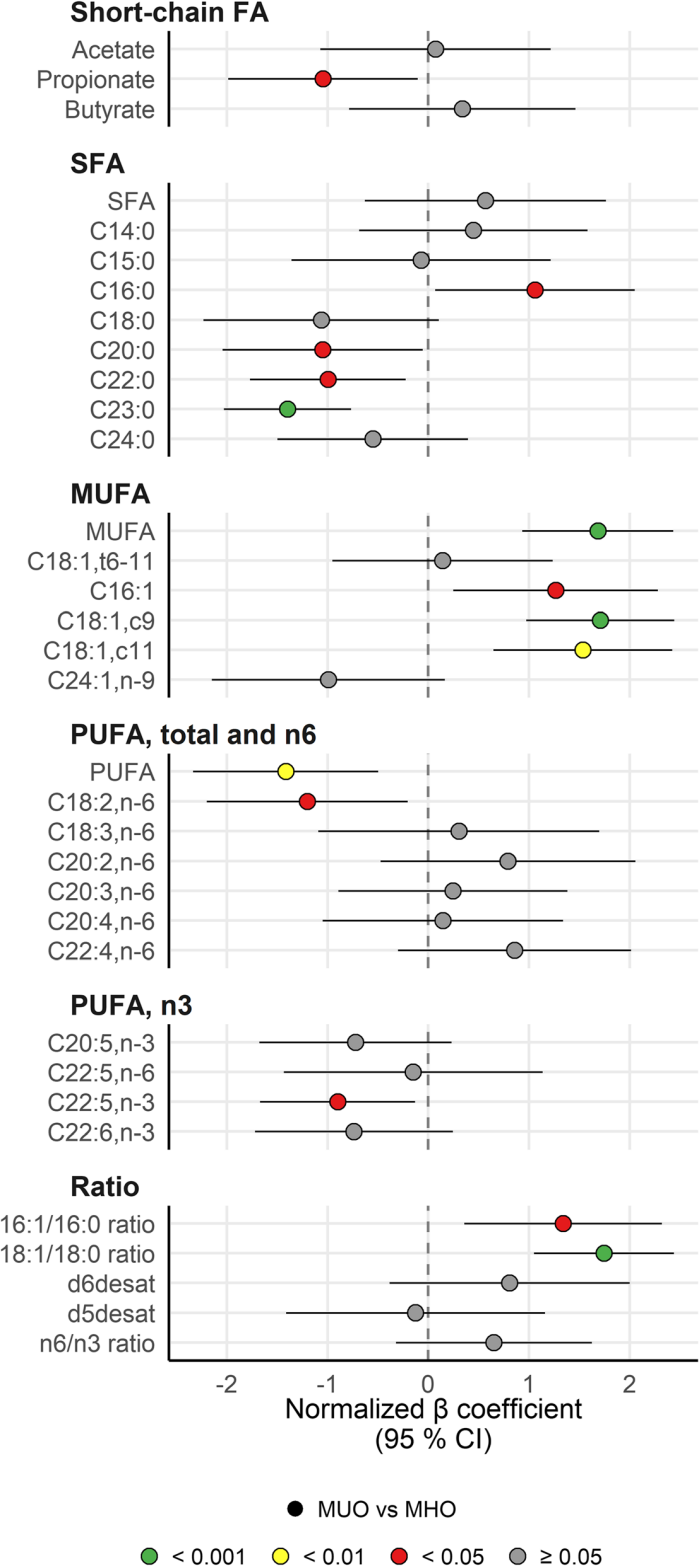
MUFAs were higher, whereas other fatty acids were lower in MUO subjects*.* The forest plot displays the β regression coefficients (mean difference) and 95% confidence interval for MUO vs MHO subjects (circles). Estimates on the right and left side of the zero-line translates to *higher* and *lower* than MHO subjects, respectively. Color denotes nominal significance level. Abbreviations: 16:1/16:0 ratio, Ratio of palmitoleic acid to palmitic acid; 18:1/18:0 ratio, Ratio of oleic acid to stearic acid; Acetate, Acetate; Butyrate, Butyrate; C14:0, Myristic acid; C15:0, Pentadecylic acid; C16:0, Palmitic acid; C16:1, Palmitoleic acid; C18:0, Stearic acid; C18:1,c11, NA; C18:1,c9, Oleic acid; C18:1,t6–11, Vaccenic acid; C18:2,n-6, Linoleic acid (LA); C18:3,n-6, Gamma-Linolenic acid (GLA); C20:0, Arachidic acid; C20:2,n-6, Dihomolinoleic acid; C20:3,n-6, Dihomo-γ-linolenic acid; C20:4,n-6, Arachidonic acid (AA); C20:5,n-3, Eicosapentaenoic acid (EPA); C22:0, Behenic acid; C22:4,n-6, Adrenic acid (AdA); C22:5,n-3, Docosapentaenoic acid (DPA); C22:5,n-6, Docosapentaenoic acid (Osbond acid); C22:6,n-3, Docosahexaenoic acid (DHA); C23:0, Tricosylic acid; C24:0, Lignoceric acid; C24:1,n-9, Nervonic acid; d5desat, Delta 5-desaturase; d6desat, Delta 6-desaturase; MHO, Metabolically healthy obese subjects; MUFA, Monounsaturated fatty acids; MUO, Metabolically unhealthy obese subjects; n6/n3 ratio, NA; Propionate, Propionate; PUFA, Polyunsaturated fatty acids; SFA, Saturated fatty acids

Fatty acids are important signaling molecules and are at the core of obesity-related diseases [13, 14]. To expand our understanding of the role of fatty acids and lipids in the MHO phenotype, we characterized the fasting plasma fatty acid profile in the two groups with obesity. The fatty acid profile in MHO was overall more favorable compared with MUO (Fig. 3). MUO had significantly lower levels of total polyunsaturated fatty acids (PUFA) and 18:2n6 and higher levels of total monounsaturated fatty acids (MUFA), 16:1, 18:1c9 and 18:1c11 than MHO. Although 16:0 was significantly higher, other long-chain saturated fatty acids (SFA) (20:0, 22:0, 23:0) were lower in MUO than in MHO.

Stearoyl-CoA desaturase (SCD) is the rate limiting enzyme in de novo lipogenesis of 16:1 and 18:1 fatty acids, in which the liver and adipose tissue are the principal sites of action. These MUFA are the major components of PL, TG and CE, and fatty acid product-to-precursor ratios have been used as an in vivo measure of desaturase activity [15–17]. Estimated SCD activity was calculated as 16:1/16:0 (SCD16) and 18:1/18:0 (SCD18) ratios. Both the SCD16 ratio and the SCD18 ratio were significantly higher in MUO compared with MHO. Estimated delta-6-desaturase (20:3n6/18:2n6) and delta-5-desaturase (20:4n6/20:3n6) activity did not differ between the groups. The SCFA acetate (2:0), propionate (3:0), and butyrate (4:0) are mainly obtained from gut microbiota fermentation. We found that MUO had significantly lower propionate levels in plasma than MHO, while butyrate and acetate did not differ between the groups (Fig. 3).

**Fig. 4:**
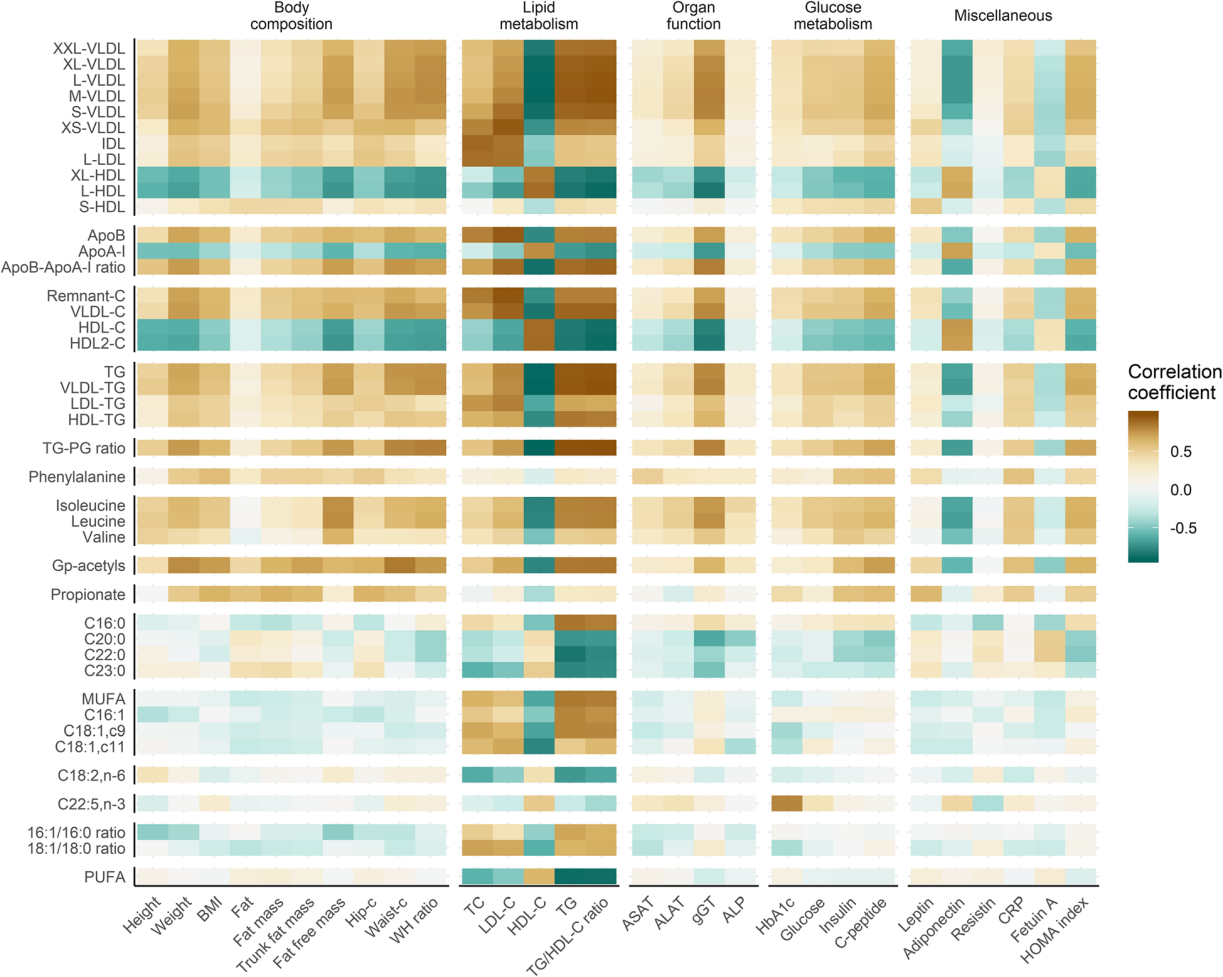
Biological markers differentially regulated in MUO, MHO and NW subjects associate with a number of clinical variables, especially body composition- and lipid-related*.* The heatmap displays Spearman’s rho (*ρ*) correlation coefficient for clinical variables (x axis) vs significant variables (y axis), as seen in Figs. 1, 2 and 3. Correlations were calculated using all three groups combined. Abbreviations: 16:1/16:0 ratio, Ratio of palmitoleic acid to palmitic acid; 18:1/18:0 ratio, Ratio of oleic acid to stearic acid; ALAT, Alanine aminotransferase; ALP, Alkaline phosphatase; ApoA-I, Apolipoprotein A-I; ApoB-ApoA-I ratio, Ratio of apolipoprotein B to apolipoprotein A-I; ApoB, Apolipoprotein B; ASAT, Aspartate aminotransferase; BMI, Body mass index; C16:0, Palmitic acid; C16:1, Palmitoleic acid; C18:1,c11, NA; C18:1,c9, Oleic acid; C18:2,n-6, Linoleic acid (LA); C20:0, Arachidic acid; C22:0, Behenic acid; C22:5,n-3, Docosapentaenoic acid (DPA); C23:0, Tricosylic acid; CRP, C-reactive protein; gGT, gamma-Glutamyltransferase; Gp-acetyls, Glycoprotein acetyls, mainly a1-acid glycoprotein; HbA1c, Glycated hemoglobin A1c; HDL-C, HDL cholesterol; HDL-C, Total cholesterol in HDL; HDL-TG, Triglycerides in HDL; HDL, high-density lipoprotein; HDL2-C, Total cholesterol in HDL2; Hip-c, Hip circumference; IDL, Intermediate-density lipoprotein; Isoleucine, Isoleucine; L, Large; LDL-C, LDL cholesterol; LDL-TG, Triglycerides in LDL; LDL, low-density lipoprotein; Leucine, Leucine; M, Medium; MHO, Metabolically healthy obese subjects; MUFA, Monounsaturated fatty acids; MUO, Metabolically unhealthy obese subjects; Phenylalanine, Phenylalanine; Propionate, Propionate; PUFA, Polyunsaturated fatty acids; NW, normal weight subjects; Remnant-C, Remnant cholesterol (non-HDL, non-LDL -cholesterol); S, Small; TC, Total cholesterol; TG-PG ratio, Ratio of triglycerides to phosphoglycerides; TG, Serum total triglycerides; TG, Triglycerides; Valine, Valine; VLDL-C, Total cholesterol in VLDL; VLDL-TG, Triglycerides in VLDL; VLDL, Very low-density lipoprotein; Waist-c, Waist circumference; WH ratio, Waist-hip ratio; XL, Extra-large; XS, Extra-small; XXL, Extremely large

We further investigated to what extent the observed differences in metabolites related to the clinical data used to categorize the subgroups initially. Overall, the lipoproteins and amino acids correlated with all clinical data (upper rectangle in Fig. 4), while the association was weaker for the fatty acids (lower rectangle in Fig. 4). However, the clinical lipid parameters (total cholesterol, LDL-cholesterol, HDL-cholesterol and TG/HDL-cholesterol ratio) showed the strongest association with all metabolites. In addition to the expected association with lipoprotein subclasses, there was an association with TG/PG ratio, amino acids, GP-acetyls, and fatty acids. We have previously shown that the systemic concentration of the liver enzyme gamma-glutamyltransferase (gGT) is different in MHO and MUO [10]. In the present study, we found that gGT was associated with the lipoprotein subclasses and amino acids. Although weak, there was also an association between gGT and SFA. However, while there was a positive association with 16:0, the long-chain SFA (20:0, 22:0, 23:0) were all negatively associated with gGT. This pattern was also reflected for the other liver markers and the SFA. HbA1c, on the other hand, showed a positive correlation with the long chain PUFA 22:5n3, but none of the other fatty acids. Adiponectin, but not resistin and leptin, negatively correlated to the BCAA, Gp-acetyls and lipoprotein subclasses except for HDL. While fat free mass, waist and waist-to-hip ratio were positively associated with VLDL, IDL and LDL subclasses, and negatively associated with HDL subclasses, there was no association with waist (Fig. 4).

### Diet

The MHO phenotype could possibly be explained by lifestyle, for example that some individuals with obesity eat a healthier diet than others. However, we found no differences in energy percent (E %), grams or kilojoule of total energy intake, fat, protein, carbohydrates or alcohol between the MHO and MUO; also, there were no differences in specific aggregated food groups (Additional figure 4).

### MHO as an intermediate-stage risk group between MUO and NW

**Fig. 5:**
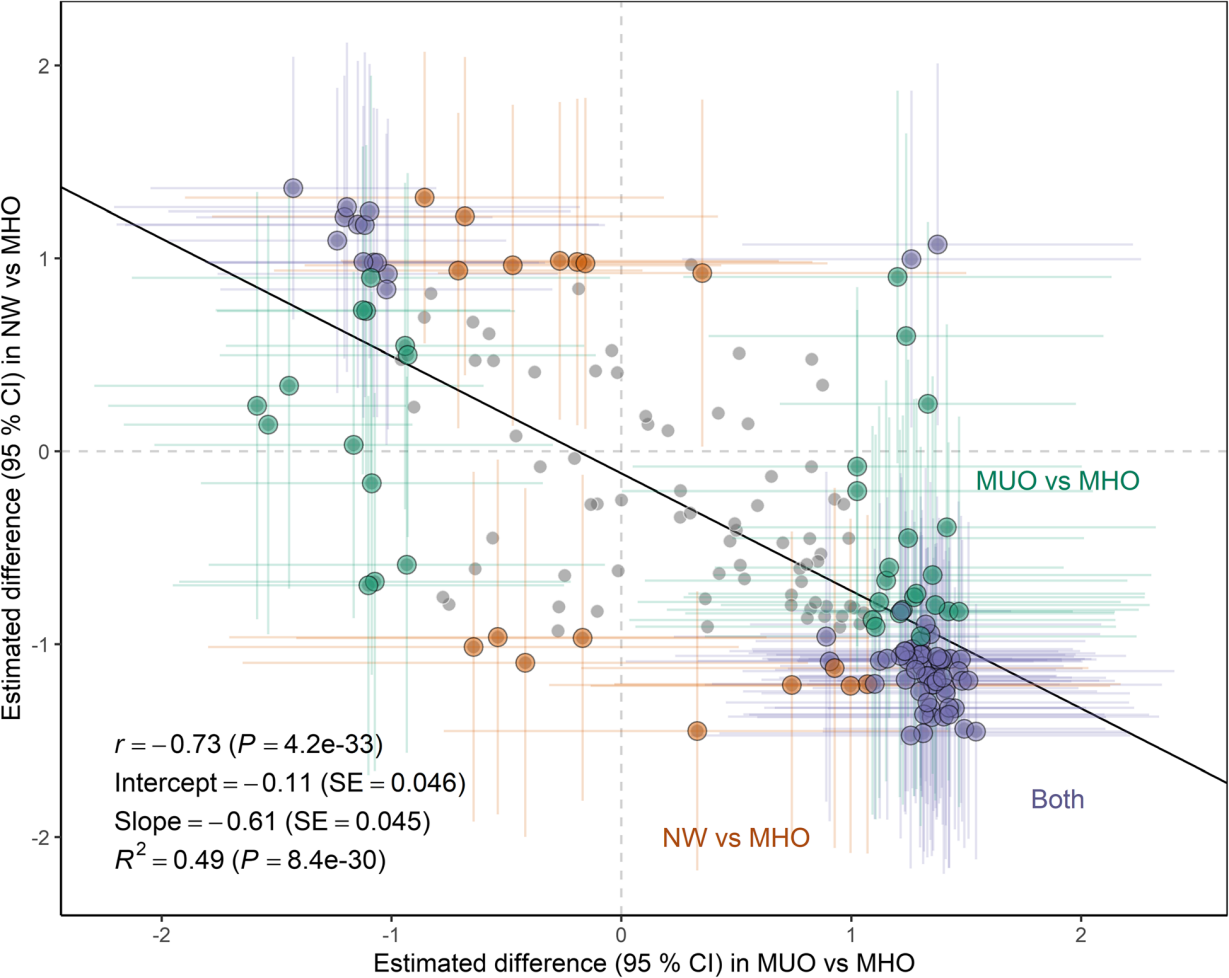
Biomarkers generally go in opposite direction in MUO and NW subject, compared with MHO subjects*.* The correlation plot displays the bivariate distribution between β regression coefficients (mean difference) and 95% confidence interval for all variables under study, for MUO vs MHO subjects (x axis) and NW vs MHO subjects (y axis). In this figure, significance level cut-off is set to *P* < 0.01. Non-significant variables are grey; those significantly different for MUO vs MHO are green; those significantly different for NW vs MHO are orange; those significantly different in both comparisons are in purple. The correlation coefficient and regression line indicates that biomarkers generally go in opposite direction in MUO and NW subject, compared with MHO subjects, with some exceptions in the upper right and lower left quadrant. Abbreviations: MUO, Metabolically unhealthy obese subjects; MHO, Metabolically healthy obese subjects; NW, Normal weight subjects; *r*, Spearman’s correlation coefficient; *R*^2^, Explained variance; SE, Standard error

To examine whether the MHO phenotype robustly corresponds to a midpoint between the MUO and NW phenotypes, we associated the respective group effect estimates for all variables where we had data for all three groups (Fig. 5). Indeed, biomarkers generally went in the opposite direction in MUO and NW, compared with MHO. Markers that were higher in MUO were generally lower in NW, and opposite, with some exceptions.

## Discussion

In the present study, using a comprehensive metabolic profiling approach, we report a panel of plasma biomarkers that associated with the degree of obesity-related clinical sequelae that make up the MUO and MHO subtypes. Our findings support that individuals with MHO phenotype have an intermediate-stage of CVD risk profile that is between MUO and NW phenotypes.

In the FINRISK cohort study, all VLDL, IDL, and LDL subclasses were associated with higher risk of future cardiovascular events, whereas the L- and M-HDL subclasses were associated with lower risk [18]. This is in line with our study, where NW had lower concentration and MUO higher concentration of all VLDL, IDL and LDL subclasses compared with MHO. Traditionally, obesity has been considered to induce insulin resistance followed by hyperlipidemia. However, another hypothesis suggested that hyperlipidemia (elevated fasting and post-prandial plasma VLDL remnants) is present prior to obesity and insulin resistance [19, 20]. In our study, individuals with MUO phenotype have higher concentration of VLDL and VLDL remnant particles compared with MHO phenotype. Furthermore, remnant particles including VLDL are synthesized with ApoB. In our study ApoB is higher in individuals with MUO and lower in NW compared with MHO. Increased levels of ApoB is positively associated with dyslipidemia and metabolic syndrome (MetS) [21] and is an important risk factor for atherosclerosis and CVD [22]. Taken together, the observed differences in VLDL, IDL and LDL subclasses and ApoB may suggest that individuals with MHO have an intermediate-stage risk of CVD compared with NW and MUO.

We found no differences in plasma levels of sphingomyelins and phosphatidylcholine among the three different subgroups. Others have measured this in visceral adipose tissue and found higher levels of sphingomyelins and sphingolipids ceramides in MUO compared with MHO and NW [23]. Serum levels of sphingomyelin and ceramide species with distinct saturated acyl chains were associated with obesity and correlated with insulin sensitivity, liver function and atherogenic dyslipidemia [24]. Since we have measured total plasma levels of sphingomyelins and phosphatidylcholine, we cannot distinguish between the compositions of fatty acids on these lipids, but this is something to explore further in future studies.

Amino acids, and in particular BCAA, are associated with obesity and risk of T2D [25, 26]. We found that the BCAA isoleucine, leucine and valine were higher in MUO, while the aromatic amino acid phenylalanine was lower in NW, compared with MHO. In line with our results, Chen et al. demonstrated increased levels of the BCAA valine and isoleucine in MUO compared with MHO [6]. However, Kim et al. did not find any differences in amino acids between MHO and MUO [27]. This discrepancy might be due to the lack of a unison definition of MHO. Elevated BCAA, in particular isoleucine and valine, is suggested to be a metabolic signature associated with insulin resistance [26, 28]. Infusion of BCAA in humans acutely worsen insulin sensitivity [29, 30] and elevations in plasma BCAA levels can be detected in people more than 10 years before developing diabetes [31]. In our study, there was a correlation between BCAA and glucose metabolism (glucose, insulin and C-peptide). However, lipid metabolism seems to be more important, in which we observed a strong positive correlation between BCAA and TG and TG/HDL-cholesterol ratio and a negative correlation between BCAA and HDL-cholesterol. Increased concentration of BCAA have previously been found in individuals with high fasting blood glucose, dyslipidemia, or increased serum TG/HDL-cholesterol ratio [32, 33]. Increased concentration of BCAA has also been found in patients with coronary artery disease, men with risk of MetS, and healthy individuals, independent of BMI [34, 35]. In a case-control sub-study of the PREDIMED trial, circulating BCAA concentration was positively associated with CVD [36]. Phenylalanine was recently identified as a novel predictor of incident heart failure hospitalization in the PROSPER-trial and the FINRISK 1997-study [37]. High levels of phenylalanine have been observed in individuals with obesity [26, 38] and phenylalanine has been identified as an important metabolite distinguishing MUO from MHO [6]. Even though individuals with NW had lower levels than MHO did, we did not find differences in phenylalanine between MUO and MHO. In line with previous studies, our results indicate that amino acid metabolism is differently regulated in people with normal weight and obesity however, there are also differences according to obesity phenotype.

Obesity and cardio-metabolic disorders associate with chronic low-grade inflammation [39]. We found no difference in CRP levels between the groups with obesity (adjusted for age and sex), although NW had lower levels of CRP compared with MHO. However, the inflammatory marker glycoprotein acetyls was decreased in NW compared with MHO, while there were no differences between MHO and MUO. The concentration of glycoprotein acetyls reflects the amount of *N*-acetyl groups in circulating glycoproteins involved in acute-phase inflammatory responses. Glycoprotein acetyls is associated with different inflammatory markers (such as IL-6, TNFa, fibrinogen and CRP) and are considered a biomarker of systemic inflammation and subclinical vascular inflammation [40]. Lawler et al. quantified glycoprotein acetyls in the Women’s Health Study and found a positive association with longitudinal risk of all-cause, cardiovascular and cancer mortality risk in initially healthy women [41]. These results suggest that obesity-related inflammation is present in both MHO and MUO phenotypes.

Serum fatty acid composition is shown to be associated with obesity [42]. Total plasma MUFA was higher and total PUFA and linoleic acid (18:2n6) were lower in the MUO compared with MHO. These results are in line with the FINRISK study [18] where they found that higher plasma levels of MUFA were associated with increased cardiovascular risk, while higher omega-6 fatty acids and docosahexaenoic acid (DHA) levels were associated with lower risk. They concluded that low PUFA and high MUFA levels are biomarkers for future cardiovascular risk [18]. In the NHANES-study, they found that high plasma concentrations of SFA and MUFA were associated with elevated HbA1c and fasting plasma glucose levels [43]. In our study there was a positive correlation between HbA1c and 22:5n3. Furthermore, in the PREDIMED study, they investigated the cross-sectional fatty acid profile in individuals with MetS versus non-MetS [44]. Their results showed the same pattern; subjects with MetS had higher levels of SFA (14:0 and 16:0) in plasma and lower levels of PUFA, in particular LA (18:2n6) [44].

The SCD enzyme catalyzes the synthesis of MUFA (16:1 and 18:1) from SFA (16:0 and 18:0), and the activity may be estimated by product-to-precursor ratios (SCD16 and SCD18, respectively) [15–17]. Increased estimated SCD activity has been associated with metabolic dysfunction, like insulin resistance and body fat mass in both animal and human studies [45]. We found that both SCD16 and SCD18 were increased in MUO compared with MHO. However, 18:1 is known to be more abundant in the diet and the high SCD18 in MUO may simply be a reflection of dietary differences. Estimated SCD activity was also measured in the PREDIMED study [44]; however, they found no difference in the SCD18 ratio and a higher SCD16 ratio in individuals with MetS compared to non-MetS [44]. In a study by Zhao et al., they investigated if free fatty acid ratios could predict the transition from MHO to MUO phenotype. They found that a high SCD18 ratio in MHO at baseline was predictive of a conversion to MUO after a 10 years follow-up [46]. The activity of the SCD enzyme is affected by different factors, including the diet, and PUFA have been shown to be inhibitors of the enzyme [47]. We have previously shown a negative correlation between estimated SCD18 activity and the concentration of PUFA in plasma (eicosapentaenoic acid (EPA) and DHA), and a corresponding reduction in plasma TG in NW, healthy individuals [17]. This is in line with the present results where MUO have lower levels of PUFA and higher estimated SCD activity compared with MHO.

Limitations of the present study include the low number of participants and that it was initially designed for other purposes than metabolic profiling. Also, cross-sectional studies are observational by nature and can never draw conclusions about causal relationships. Strengths of the study include detailed profiling across multiple metabolic pathways in subgroups with obesity and NW.

## Conclusions

In summary, comprehensive metabolic profiling supports that individuals with MHO phenotype have intermediate-stage cardiovascular disease risk profile compared with NW and MUO. Such a detailed profiling of obesity phenotypes may lead to earlier and more accurate identification of individuals at high cardio-metabolic disease risk, facilitating better preventive strategies.

## Supplementary information

**Additional file 1: Figure S1.** Principal component analysis separated the study groups for some, but not all, data types*.* Panels A-D display standard clinical data, Nightingale data, Vitas plasma fatty acids data, and dietary intake data, respectively, for MUO, MHO and NW subjects as labelled directly by colors. Abbreviations: MHO, Metabolically healthy obese subjects; MUO, Metabolically unhealthy obese subjects; NW, Normal weight subjects; PC, Principal component.

**Additional file 2: Figure S2.** Absolute level, but not relative level, of various lipid types are generally lower in NW and higher in MUO, compared with MHO subjects*.* The forest plot displays the β regression coefficients (mean difference) and 95% confidence interval for MUO vs MHO subjects (circles) and NW vs MHO subjects (squares). Estimates on the right and left side of the zero-line translates to *higher* and *lower* than MHO subjects, respectively. Color denotes nominal significance level. Abbreviations: HDL, High-density lipoprotein; IDL, Intermediate-density lipoprotein; L, Large; LDL, Low-density lipoprotein; M, Medium; MHO, Metabolically healthy obese subjects; MUO, Metabolically unhealthy obese subjects; NW, Normal weight subjects; S, Small; VLDL, Very low-density lipoprotein; XL, Extra-large; XS, Extra-small; XXL, Extremely large.

**Additional file 3: Figure S3.** There is systematic variation in lipid species content for 14 lipid subclasses across all study groups*.* The figure shows the distribution of lipid species across all 14 subclasses for MUO, MHO and NW groups. The left-hand side “% of total lipids” column and color-coding correspond to the absolute concentration of lipid species reported in the boxplot-dotplot columns on the right-hand side. Abbreviations: HDL, High-density lipoprotein; IDL, Intermediate-density lipoprotein; L, Large; LDL, Low-density lipoprotein; M, Medium; MHO, Metabolically healthy obese subjects; MUO, Metabolically unhealthy obese subjects; NW, Normal weight subjects; S, Small; VLDL, Very low-density lipoprotein; XL, Extra-large; XS, Extra-small; XXL, Extremely large.

**Additional file 4: Figure S4.** Dietary intake was similar for MUO and MHO subjects*.* The forest plot displays the β regression coefficients (mean difference) and 95% confidence interval for MUO vs MHO subjects (circles) and NW vs MHO subjects (squares). Estimates on the right and left side of the zero-line translates to *higher* and *lower* than MHO subjects, respectively. Color denotes nominal significance level. Abbreviations: CHO, Carbohydrate; E%, Percent of total energy intake; g, Grams; kJ, Kilojoule; MHO, Metabolically healthy obese subjects; MUO, Metabolically unhealthy obese subjects.

## Data Availability

The datasets used and/or analysed during the current study are available from the corresponding author on reasonable request.

## Abbreviations

BCAA: Branched chain amino acid
BMI: Body mass index
CE: Cholesteryl ester
CRP: C-reactive protein
CVD: Cardiovascular disease
DHA: Docosahexaenoic acid
EPA: Eicosapentaenoic acid
FC: Free cholesterol
gGT: gamma-glutamyltransferase
HDL: High density lipoprotein
HOMAir: Homeostasis model assessment of insulin resistance
IDL: Intermediate density lipoprotein
L: Large
LDL: Low density lipoprotein
M: Medium
MetS: Metabolic syndrome
MHO: Metabolically healthy obesity
MOU: Metabolically unhealthy obesity
MUFA: Monounsaturated fatty acid
NW: Normal weight
PCA: Principal component analyses
PL: Phospholipid
PUFA: Polyunsaturated fatty acid
S: Small
SCD: Stearoyl CoA desaturase
SCFA: Short chain fatty acid
SFA: Saturated fatty acid
TG: Triglyceride
T2D: Type 2 diabetes
VLDL: Very low density lipoprotein
XL: Extra-large
XXL: Extremely large
XS: Extra-small
