# Supplementary figures and images for: A comprehensive metabolic profiling of the metabolically healthy obesity phenotype
Source: Lipids Health Dis. 2020 May 9;19:90. doi: 10.1186/s12944-020-01273-z (PMC7211343; doi:10.1186/s12944-020-01273-z)

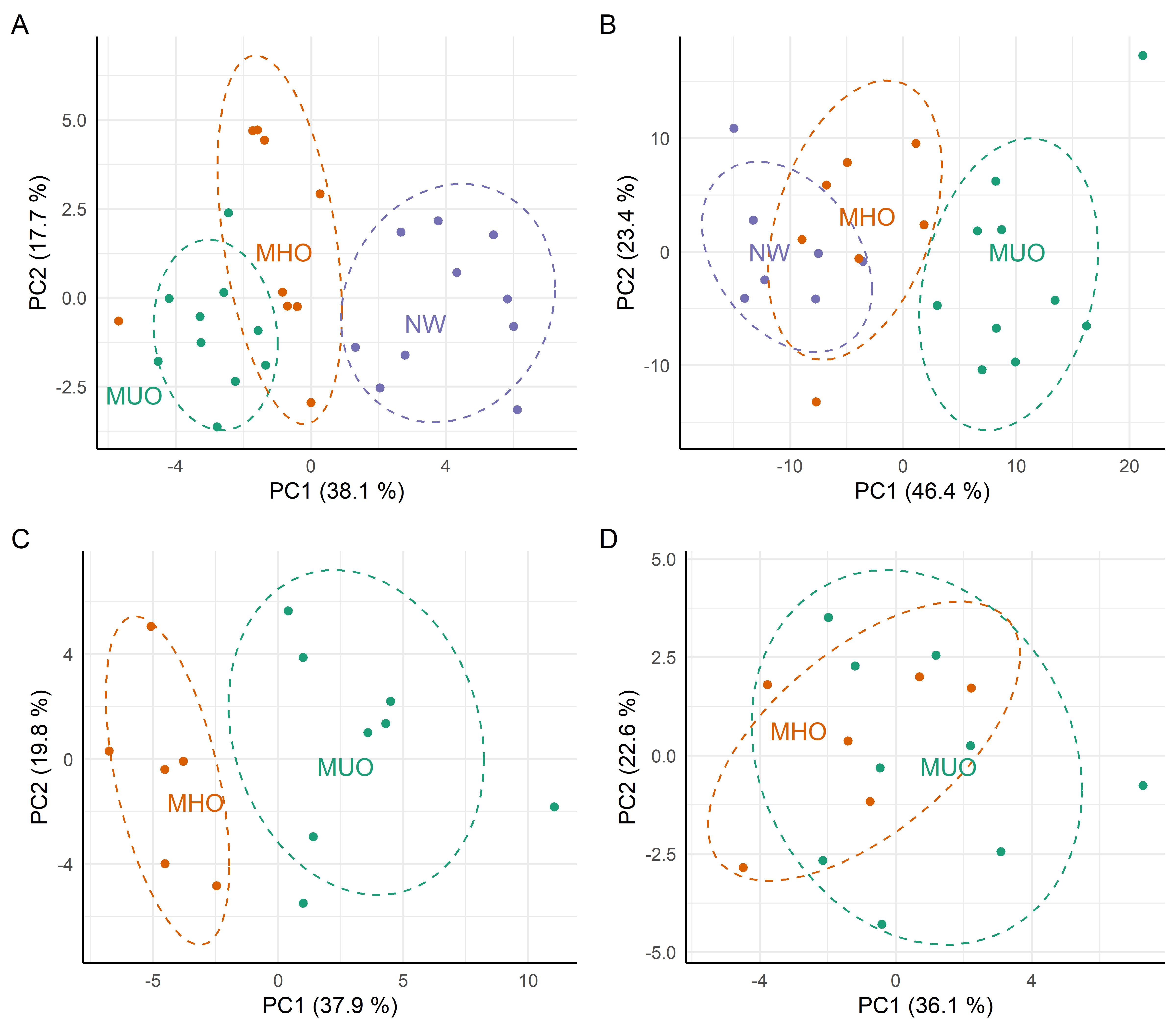

Supplement: Supplementary file 1 — Additional file 1: Figure S1. Principal component analysis separated the study groups for some, but not all, data types. Panels A-D display standard clinical data, Nightingale data, Vitas plasma fatty acids data, and dietary intake data, respectively, for MUO, MHO and NW subjects as labelled directly by colors. Abbreviations: MHO, Metabolically healthy obese subjects; MUO, Metabolically unhealthy obese subjects; NW, Normal weight subjects; PC, Principal component. [file 12944_2020_1273_MOESM1_ESM.png]

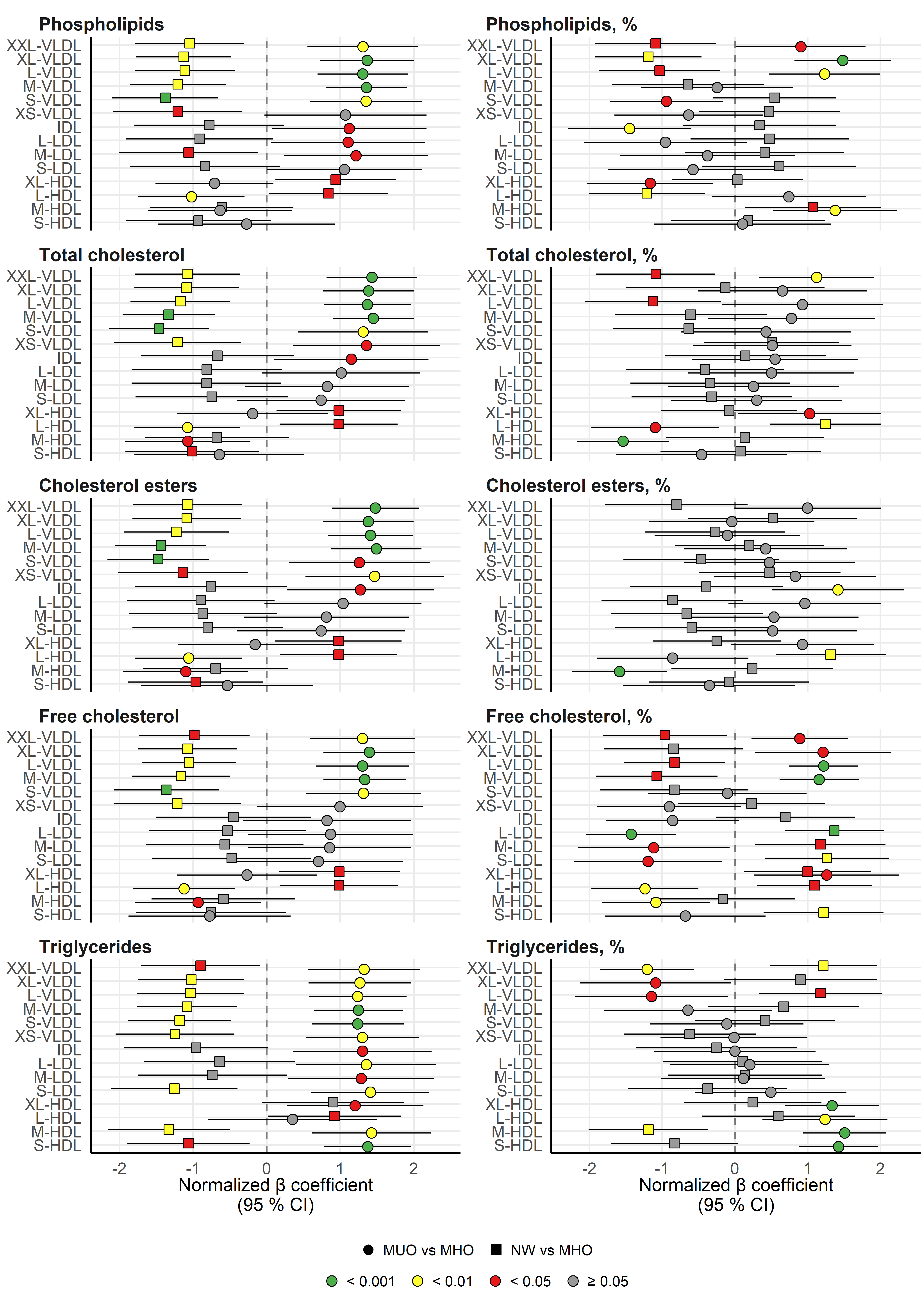

Supplement: Supplementary file 2 — Additional file 2: Figure S2. Absolute level, but not relative level, of various lipid types are generally lower in NW and higher in MUO, compared with MHO subjects. The forest plot displays the β regression coefficients (mean difference) and 95% confidence interval for MUO vs MHO subjects (circles) and NW vs MHO subjects (squares). Estimates on the right and left side of the zero-line translates to higher and lower than MHO subjects, respectively. Color denotes nominal significance level. Abbreviations: HDL, High-density lipoprotein; IDL, Intermediate-density lipoprotein; L, Large; LDL, Low-density lipoprotein; M, Medium; MHO, Metabolically healthy obese subjects; MUO, Metabolically unhealthy obese subjects; NW, Normal weight subjects; S, Small; VLDL, Very low-density lipoprotein; XL, Extra-large; XS, Extra-small; XXL, Extremely large. [file 12944_2020_1273_MOESM2_ESM.png]

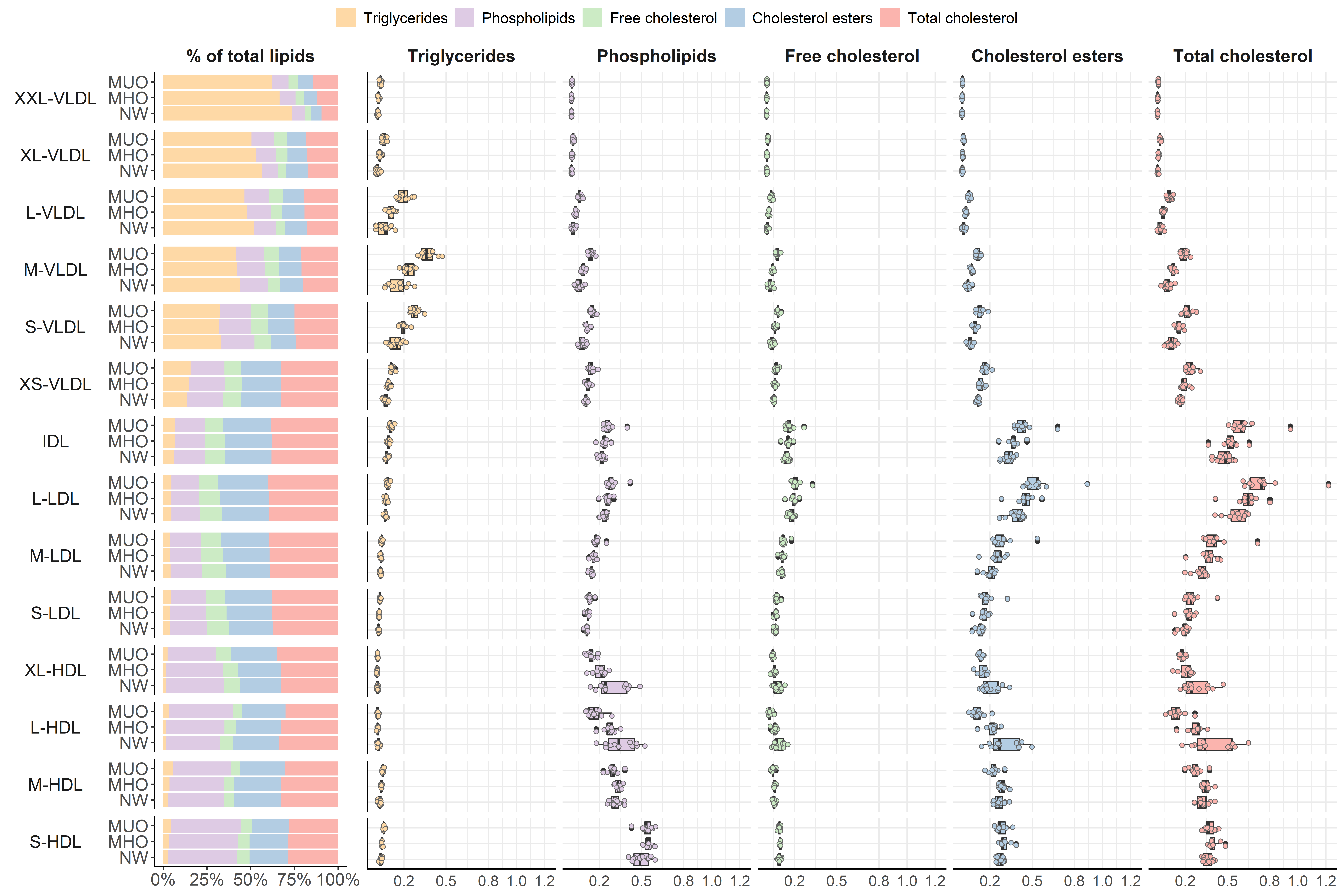

Supplement: Supplementary file 3 — Additional file 3: Figure S3. There is systematic variation in lipid species content for 14 lipid subclasses across all study groups. The figure shows the distribution of lipid species across all 14 subclasses for MUO, MHO and NW groups. The left-hand side “% of total lipids” column and color-coding correspond to the absolute concentration of lipid species reported in the boxplot-dotplot columns on the right-hand side. Abbreviations: HDL, High-density lipoprotein; IDL, Intermediate-density lipoprotein; L, Large; LDL, Low-density lipoprotein; M, Medium; MHO, Metabolically healthy obese subjects; MUO, Metabolically unhealthy obese subjects; NW, Normal weight subjects; S, Small; VLDL, Very low-density lipoprotein; XL, Extra-large; XS, Extra-small; XXL, Extremely large. [file 12944_2020_1273_MOESM3_ESM.png]

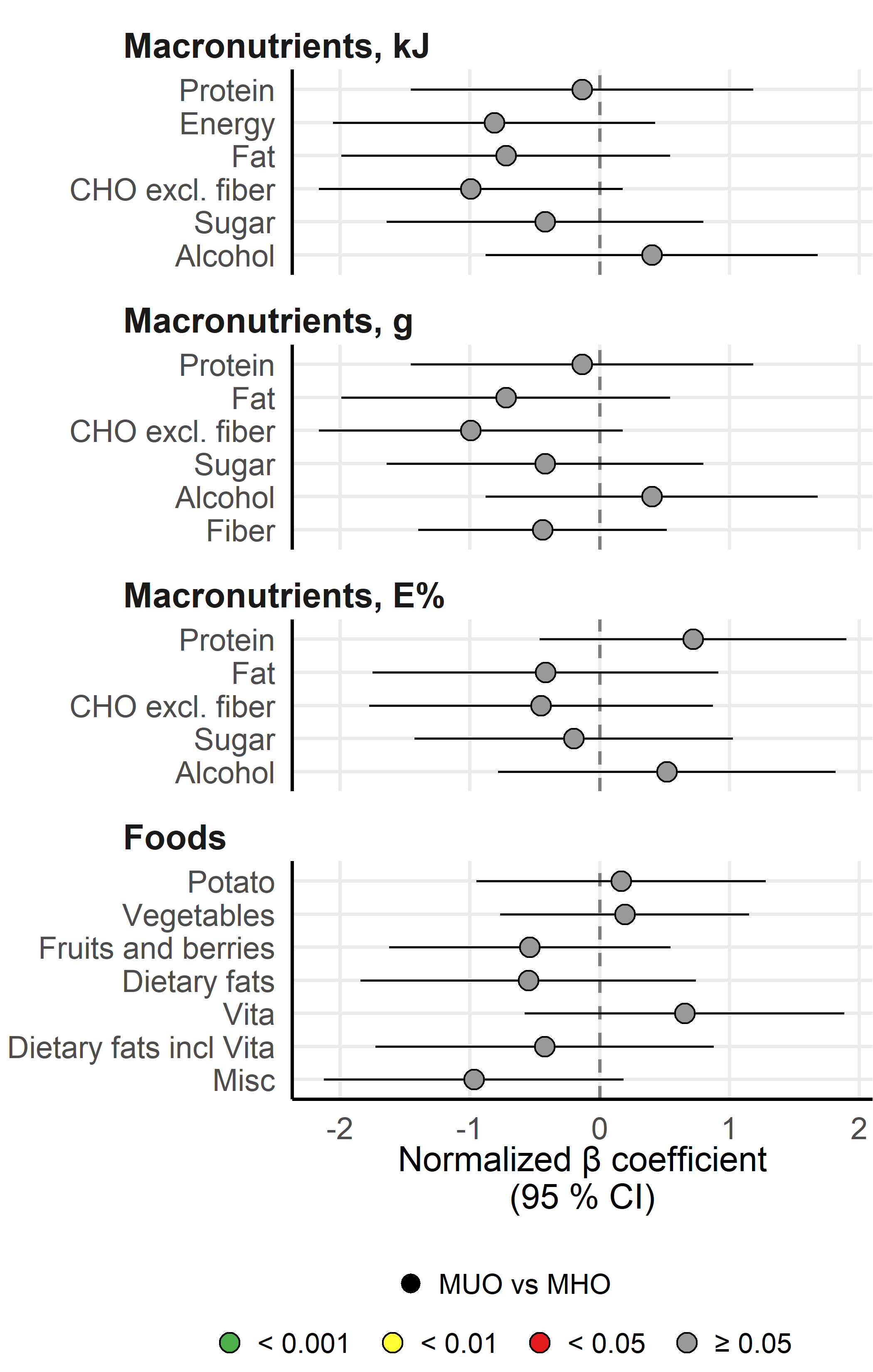

Supplement: Supplementary file 4 — Additional file 4: Figure S4. Dietary intake was similar for MUO and MHO subjects. The forest plot displays the β regression coefficients (mean difference) and 95% confidence interval for MUO vs MHO subjects (circles) and NW vs MHO subjects (squares). Estimates on the right and left side of the zero-line translates to higher and lower than MHO subjects, respectively. Color denotes nominal significance level. Abbreviations: CHO, Carbohydrate; E%, Percent of total energy intake; g, Grams; kJ, Kilojoule; MHO, Metabolically healthy obese subjects; MUO, Metabolically unhealthy obese subjects. [file 12944_2020_1273_MOESM4_ESM.png]
